# Clinical Competence in ST-segment Elevation Myocardial Infarction Management by Recently Graduated Physicians Applying for a Medical Residency Program

**DOI:** 10.36660/abc.20180309

**Published:** 2020-01

**Authors:** Ugo Stocco Aimoli, Carlos Henrique Miranda

**Affiliations:** 1 Universidade de São Paulo - Campus de Ribeirão Preto - Divisão de Medicina de Emergência do Departamento de Clínica Médica, Ribeirão Preto, SP - Brazil

**Keywords:** ST Elevation Myocardial Infarction, Medical Staff, Hospital, Education, Medical, Clinical Competence, Internship and Residence

## Abstract

**Background:**

A significant reduction in the morbidity and mortality related to ST-segment elevation myocardial infarction (STEMI) has been achieved with the development of reperfusion therapies. Early diagnosis and correct initial management are important to ensure this benefit. In Brazil, recent graduates in medicine are responsible for a large part of the initial care provided for these patients.

**Objective:**

To assess the clinical competence in the diagnosis and initial treatment of STEMI by newly graduated physicians applying for a medical residency program.

**Methods:**

We assessed the performance of 771 applicants for the direct entry selection process of the FMRP-USP Clinical Hospital Medicine Residency Program, performed in a simulated setting of STEMI, with professional actors and medical evaluators, using a standardized checklist following the recommendations of the Brazilian Guidelines for the management of this disease.

**Results:**

The general performance score presented a median of 7 and an interquartile range of 5.5-8.0. In relation to the items assessed: 83% required ECG monitoring, 57% requested the insertion of a peripheral venous access catheter, 95% administered acetylsalicylic acid, 80% administered a second antiplatelet agent (p2y12 inhibitor), 66% administered nitrate, 71% administered morphine, 69% recognized the diagnosis of STEMI, 71% assessed the pain duration, 63% recognized the need for immediate transfer, 34% showed adequate communication skills and only 25% insisted on the transfer even in case of non-availability of beds.

**Conclusions:**

The initial diagnosis and management of STEMI need to be improved in medical undergraduate courses and inserted into the reality of the hierarchical network structure of the Brazilian Unified Health System (SUS).

## Introduction

Over the last decades there has been a great development in the treatment of ST-segment Elevation Myocardial Infarction (STEMI) with an important reduction in its morbidity and mortality, especially with the emergence of fibrinolytic therapy and primary angioplasty procedures which allow for immediate recanalization of the involved coronary artery.^1^

However, to reap these benefits, it is important to make an early diagnosis of STEMI, using a resting ECG and to organize the referral of these patients to tertiary centers capable of carrying out reperfusion therapy.^2^

Inside the Brazilian Unified Health System (SUS), which is categorized into levels of care, this patient is usually assisted in Emergency Care Units (UPAs). Because of the lack of professional doctors dedicated exclusively to emergency care, a great deal of this healthcare is provided by recently graduated physicians.^3^ The lower clinical experience of these professionals can compromise the diagnosis and initial management of these patients in this critical period, in which agility makes all the difference.^4^

The objective of this study was to assess the clinical competence for the diagnosis and initial management of STEMI by recently graduated physicians applying for a medical residency program.

## Methods

**Figure 1:**
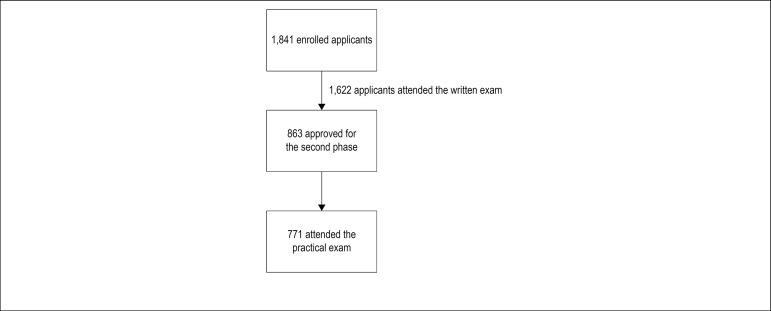
Flowchart with the number of applicants in the different phases of the medical residency selection process.

This is a single-centered cross-sectional study, which used simulation with professional actors, organized within the objective structured clinical examination system, better known as OSCE, as the evaluation method to verify the clinical competencies of recently graduated physicians in STEMI management. ^5^ A scenario of initial patient treatment in an emergency care unit (UPA) was set up. This scenario was designed both for research data collection and as one of the practical exam stations of the direct entry public selection process of the Ribeirão Preto Medical School Clinics Hospital of the University of São Paulo (HC-FMRP-USP), for admission of first-year residency physicians to different specialties, in 2016. The practical exam to which this study refers was performed only by the applicants approved in the first-stage written exam, consisting of multiple choice questions, at a rate of 4 candidates per vacancy (except for the Infectology and Neurosurgery fields, where the proportion was of 5 candidates per vacancy). A total 1,841 candidates applied for the first-stage exam, out of whom 1,622 performed the written exam (first stage), 863 applicants were approved for the practical exam (second stage) and 771 applicants actually took the practical exam in the STEMI station (Figure 1).

### The scenario organization

Before beginning the treatment, the applicants were informed that they would assist an adult patient in a UPA. The scenario was organized as a standard doctor’s office, where a properly trained actor (Appendix B)* was lying on a stretcher and the evaluator was sitting at a table connected to a computer which displayed the checklist of predetermined competencies and attitudes. Inside the room, there was a printed instruction on the wall (Appendix A)* saying that the patient in question had been admitted with a complaint of chest pain, and that the nurse prioritized his care and traced a 12-lead ECG. An ECG with typical tracing for anterior wall STEMI was provided for the applicant (Appendix F)*. Some history and physical exam data were previously provided (severe retrosternal pain with an intensity 8/10 of sudden onset, associated with nausea, no irradiation and other associated symptoms, no other health problems, no use of medication in the previous 24 hours, physical examination revealed an oxygen saturation level of 98% on room air, a blood pressure of 130/80 mmHg and a heart rate of 88 bpm and no need for physical examination, considering the other data were normal). On the printed instructions, there were two tasks to be performed by the applicants: first, to define the main diagnosis for the evaluator; second, to provide the most adequate clinical management for the patient. The applicant was given a deadline of five minutes to execute these tasks. Time was clocked using an automated system. The actor was trained to ask the applicant two active questions in a row, only when the applicant suggested the need for a transfer to a hospital or Cardiology Center: “Doctor, why do I need to go to a hospital?”. And after 10 seconds: “Doctor, what if there is no room?”.

In the consulting room, there was a list with all the medications and procedures available in that UPA (Appendix E)*, and there were no fibrinolytic therapy and coronary angioplasty available on site. A total of 11 simulated doctor offices were organized, which worked simultaneously during the evaluation.

### The evaluators’ training for filling out the checklist

Sixteen evaluators, all experienced doctors, were trained to fill out the checklist electronically , using a computer available in the scenario, simultaneously with the execution of the tasks by the applicants. The evaluators were instructed not to communicate with the applicants, who should only follow the written instructions. The instructions used in the evaluators’ training are available in Appendix C* of the supplementary appendix. Each applicant was evaluated by only one examiner. To reduce heterogeneity between the examiners a training was performed immediately before the beginning of the practical station, when each item of the checklist was reviewed in terms of possible answers. There was an external evaluator who was positioned in the access passage to all simulated consulting rooms and centralized any interpretation doubts concerning the applicants’ answers. This external evaluator’s opinion was always accepted in cases of doubtful interpretation on the part of the evaluators.

### The development of the checklist

The checklist was developed by a cardiologist with experience in treating STEMI patients following the V Guideline of the Brazilian Society of Cardiology on Acute Myocardial Infarction Treatment with ST-Segment Elevation and revised by other five experienced physicians.^6^ The checklist included 12 different items, six of which were directly concerned with the treatment (0.5 points each, with a total of 3.0 points): requirement of ECG monitoring, insertion of a peripheral venous access catheter, acetylsalicylic acid administration, administration of a second antiplatelet agent (p2y12 inhibitor), sublingual nitrate administration and morphine administration (it was not necessary to specify the correct dosage of these medications, but only to mention them). And the six remaining items were directly associated with the diagnosis and transfer management: recognition of the diagnosis of STEMI (2.0 points), assessment of pain duration, which is an important aspect in the determination of the therapeutic conduct in this case (1.0 point), recognition of immediate need for patient transfer (2.0 points) in order to perform reperfusion therapy (thrombolytic or primary angioplasty), one item related with communication skills towards the patient, explaining the disease and the treatment using simple and objective language (1.0 point) and one item related with reinforcing the need to transfer the patient for a tertiary service, even in case of non-availability of beds (1.0 point), defined as “zero vacancy”, according with the Resolution 2077/14 of the Federal Medicine Council (CFM) for urgencies and emergencies. Finally, there was one item of negative evaluation, which consisted of delaying treatment to wait for myocardial necrosis markers (a two-point reduction in the score). More details on the checklist can be found in Appendix C* of the supplementary appendix. The score ranged from 0 to 10 points, being directly proportional to the clinical competence observed in the simulated scenario approach. According to the organization of the checklist, the most important items were: identification of STEMI diagnosis (2.0 points) and immediate transfer to reperfusion (2.0 points). In relation to the correct diagnosis, it was necessary to specify ST-segment elevation, because electrocardiographic criteria for this diagnosis are quite well defined. We considered as a partial answer the identification of infarction with no reference to ST-segment elevation, since, even in such case, the applicant could initiate the recommended therapeutic conduct. Other drug therapies were included as adjuvant therapy and had the same value (0.5 points), since all of them were classified as Class-I recommendation in the most recent Brazilian guidelines for initial management of STEMI.

### Demographic and education data

These data were obtained from the application form filled out by the candidates: age, sex, graduation year, state of origin, medical school of origin. Data related to the performance in the evaluation, obtained through the electronic checklist, were exported to Excel spreadsheets from a system exclusively developed to carry out this practical assessment. This project was approved by the Ethics Committee in Research of our institution under protocol number 838/2017.

### Statistical Analysis

Sample size corresponded to all the applicants who performed the station practical exam of the selection process for the medical residency program of the HC-FMRP-USP, in 2016. Normality of distribution was checked using the Shapiro-Wilk test. Quantitative variables with normal distribution were expressed as mean (standard deviation) and the other quantitative variables as median and interquartile range. Categorical variables were expressed as percentage. Student-t test was used to compare two quantitative variables with normal distribution and Mann-Whitney test was used for comparison between two quantitative variables not normally distributed. The Chi-square test was used to compare two categorical values. A two-tailed p-value < 0.05 was considered significant. Data analyses and graphic building were performed by STATA 13.1 for Windows (STATA software, College Station, TX, USA).

## Results

The demographic characteristics of the 771 applicants who performed the tasks in this simulated station are shown in Table 1. The median age was 25 years (24-27 years), and the majority of the applicants were males (58%). Most applicants were newly formed physicians (90%), among whom 71% had graduated less than a year before and 19% had graduated between 1-2 years before. Most candidates had come from a public university (66%) in the Southeast region (65%).

**Table 1:** Demographic characteristics of the recently graduated physicians who took part in the practical examin on STEMI medical care

Characteristics		n = 771 individuals
Age (years); median (19)		25 (24-27)
Male sex; n(%)		444 (58)
**Years from graduation; n(%)**		
	< 1 year	549 (71)
	≥ 1-2 years	145 (19)
	≥ 2-3 years	44 (06)
	≥ 3-4 years	12 (01)
	≥ 4 years	21 (03)
**Institution where graduated; n(%)**		
	Public	509 (66)
	Private	254 (33)
	Foreign	8 (01)
**Region of origin; n(%)**		
	Southeast	499 (65)
	Northeast	173 (22)
	South	48 (06)
	Central-West	33 (04)
	North	10 (01)
	Abroad	08 (01)

**Figure 2:**
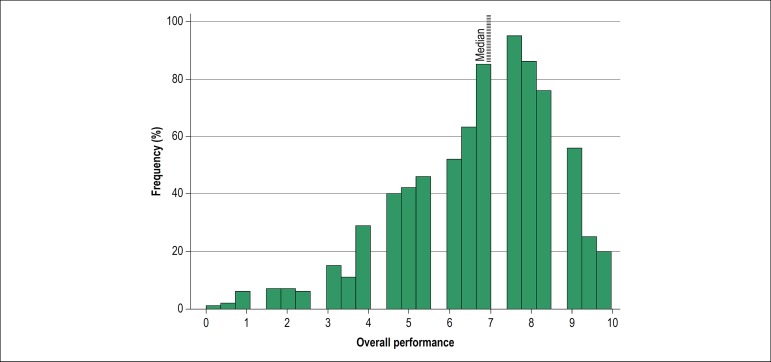
Frequency histogram showing the general score distribution obtained by the recently graduated physicians during practical assessment in the simulated station of STEMI medical care.

Regarding the general performance of the applicants, a median of 7.0 (interquartile range: 5.5-8.0) and a mean of 6.74 with a standard deviation of 1.93 were observed (Figure 2).

**Figure 3:**
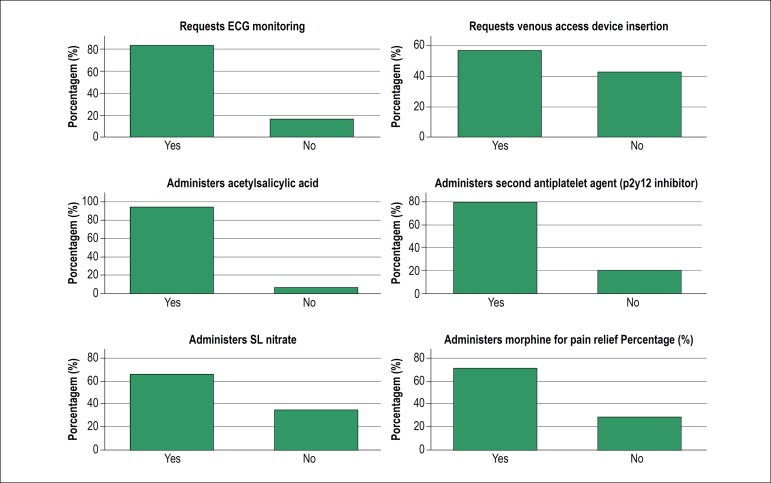
Bars graphic showing the performance of the recently graduated physicians in each of the six items of the cheklist related directly to the treatment during practical assessment in the simulated station of STEMI medical care.

In relation to each item of the checklist directly associated with the treatment, the following result was observed: 83% of the applicants requested ECG monitoring, 57% oriented the insertion of a peripheral venous access device, 95% administered acetylsalicylic acid, 80% added a second antiplatelet agent (P2Y12 inhibitor), 66% administered sublingual nitrates e 71% administered intravenous morphine for pain relief (Figure 3).

**Figure 4:**
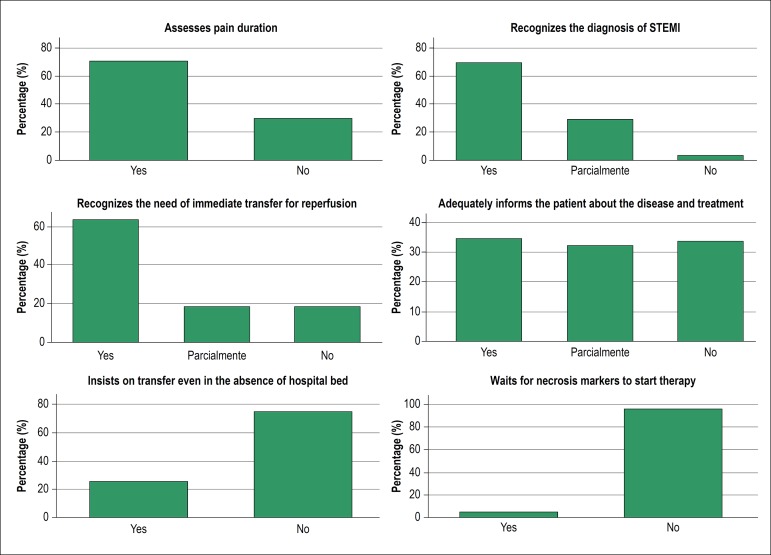
Bars graph showing performance of recently graduated physicians in each of the six items of the checklist directly related to diagnosis and management of the patient’s tranfer during practical evaluation in the simulated station of STEMI medical care.

The following proportion was observed in relation to the checklist items concerning with the diagnosis and transfer management: 69% of the applicants identified the diagnosis of STEMI and 28% identified the diagnosis of infarction, but did not mention ST-segment elevation. The duration of pain was verified by 71% of the applicants. The majority of the applicants recognized the need for transfer to perform the reperfusion procedure (63%) and 19% recognized this necessity, however they did not specify the reason for the transfer (reperfusion therapy), 18% did not raise the need for transfer. Communication skills were considered adequate in 34% of the applicants (they provided adequate explanation on disease and treatment), 32% explained only one of these items (disease or treatment) and 34% did not approach any of the two items with the patient. Only 25% of the applicants insisted on the transfer to a tertiary center even in case of non-availability of beds after the actor’s intervention. A total of 4% of the applicants kept waiting for the markers of myocardial necrosis in order to define the therapeutic conduct (Figure 4).

When comparing the applicants who performed best (general score > 7) with those with a lower performance (general score ≤7), no differences were observed in relation to the age, mean time of graduation and geographic region of origin. However, a higher prevalence of males was observed in the group with lower performance (63% vs. 52%; p = 0.003) and a higher prevalence of graduates from public universities in the group with greater performance (71% vs. 61%; p = 0.0016). When comparing their performance in each of the 12 items of the checklist, the first group presented superior results in all items. Nevertheless, the greatest differences between the groups occurred with regard to diagnosis aspects, transfer management and communication skills. The prevalence of identification of immediate transfer need was 100% vs. 31%; p < 0.0001, the percentage of adequate communication skills was 55% vs. 15%; p < 0.0001, the identification of the diagnosis of STEMI was 89% vs. 51%; p < 0.0001 and the persistence to organize the transfer even in case of non-availability of beds was 45% vs. 8%; p < 0.0001 (Table 2).

**Table 2:** Comparison between the groups with greater performance (general score > 7) and lower performance (general score ≤ 7) in the simulated practice regarding STEMI medical care

Characteristic	General score		Relative difference	p-value
	> 7 n = 363	≤ 7 n = 408		
**Demographic**				
Age, mean ± DP	26 ± 7	26 ± 5		0.99
Age, years; median(19)	25 (24-27)	25 (24-27)		
Male sex, n(%)	189 (52)	255 (63)	-11%	0.0034
**Medical Graduation**				
Time from conclusion, years; median(19)	0 (0-1)	0(0-1)		> 0,05
Private institution, n(%)	99(27)	155(38)	-11%	0.0016
Public institution, n(%)	259(71)	250(61)	+10%	0.0032
**Region of origin, n(%)**				**0.792**
Southeast	245(67)	254(62)	+5%	
Northeast	72(20)	101(25)	-5%	
South	24(07)	24(06)	+1%	
Central-West	16(04)	9(02)	+2%	
North	3(01)	7(02)	-1%	
**Checklist Items, yes; n(%)**				
Recognizes need for transfer	363(100)	125(31)	+69%	< 0.0001
Adequately informs the patient	201(55)	63(15)	+40%	< 0.0001
Recognizes STEMI	323(89)	207(51)	+38%	< 0.0001
Insists on tranfer in the absence of bed	162(45)	31(08)	+37%	< 0.0001
Requests venous access insertion	245(67)	193(43)	+24%	< 0.0001
Assesses pain duration	300(83)	244(60)	+23%	< 0.0001
Administers SL nitrate	274(75)	232(57)	+18%	< 0.0001
Administers IV morphine	293(81)	258(63)	+18%	< 0.0001
Administers P_2_Y_12 _inhibitors	318(88)	298(73)	+15%	< 0.0001
Requests ECG monitoring	318(88)	323(79)	+9%	0.0018
Administers acetylsalicylic acid	355(98)	377(92)	+6%	0.0006
Waits for necrosis markers to start therapy	00(00)	32(08)	-8%	< 0.0001

SD: standard deviation; ECG: electrocardiogram; STEMI: ST-segment elevation myocardial infarction; SL:sublingual; IV: intravenous.

When comparing the 90 candidates from our institution with all the other applicants from the remaining institutions, the following results were found: ECG monitoring requirement (86% vs. 83%; p = 0.50), insertion of peripheral venous access device (81% vs. 54%; p < 0.0001), pain duration assessment (63% vs. 71%; p = 0.11), accurate STEMI diagnosis (76% vs. 68%; p = 0.20), acetylsalicylic acid administration (100% vs. 94%; p = 0.01), second antiplatelet agent administration (80% vs. 76%; p = 0.004), nitrate administration (72% vs. 65%; p = 0.16), morphine administration (82% vs. 70%; p = 0.01), immediate transfer requirement (82% vs. 61%; p < 0.0001), adequate communication skill (49% vs. 32%; p = 0.001), transfer requirement even in case of non-availability of beds (38% vs. 23%; p = 0.003).

## Discussion

This study shows the need to enhance training, during undergraduate courses, on the identification of STEMI and, especially, to increase knowledge of the assistance flow in the care line of this disease.

According with report data on medical demography in Brazil, in 2016, there were 18,753 new registrations in Regional Medical Councils, most of them from the Southeast region (50% of the registrations).^7^ Thus, every year, there is a great number of young doctors graduating from several medical colleges in Brazil. Many of them begin medical practice by choice, or due to difficulties to enter residency programs, and even concomitantly with these programs to complement their earnings. Due to lack of professionals exclusively dedicated to emergency care, newly graduated doctors usually take shifts in the emergency department.

In Brazil, about 50% of STEMI patients are treated with reperfusion therapy,^8^ reaching a percentage of 20% in public hospitals of certain regions.^9^ In a Brazilian registry of acute coronary syndrome, in which 2,453 patients were included, high rates of acetylsalicylic acid use (97.6%) and P2Y12 inhibitor (89.5%) were observed in the first 24 hours, similar to the rates found in this study, but among STEMI patients, only 35.9% received primary angioplasty and 25.3% received fibrinolytic therapy.^10^ Several factors contribute to this reduced number of patients who receive reperfusion therapy in Brazil. However, one of these factors refers to the lack of recognition and inappropriate management by newly graduated physicians in primary care. It is unlikely that a STEMI will not be identified by a cardiologist, but a less experienced doctor may have difficulties. On the other hand, a patient with STEMI is more likely to be assisted in an Emergency Care Unit of the Brazilian National Health System (SUS) by a recently graduated doctor than by a specialist in the field. Therefore, in order to increase the proportion of patients with STEMI receiving reperfusion therapy in Brazil, it is necessary to encourage and improve the teaching of this disease during undergraduate courses in medicine.

Another study carried out in Brazil, in which 97 patients victims of acute myocardial infarction were interviewed, found that only 33% managed to get immediate hospitalization, whereas 67% of the patients went through up to five units until they could be admitted. Among the main reasons for the lack of hospitalization was the fact that the physician referred the patient back home due to no identification of this diagnosis.^11^ Ghanem et al.,^12^ using standard questionnaires applied to medical students, found that only 22% of them knew how to aid patients with myocardial infarction.^12^ Little et al.^13^ *s*howed that only 61% of the students in the last year of medical training recognized the electrocardiographic diagnosis of inferior wall STEMI.^13^

Realistic simulation was the methodology employed in this assessment. This is an important assessment tool of health professionals, because it enables the performance of different evaluations within the same systematic framework, ensuring uniformity, which would be impracticable in a real scenario. On the other hand, it tries to reproduce directly the environment where this assistance usually occurs, allowing for knowledge, skills and attitude assessment.^14^ We consider this simulation as highly reliable, since it was performed inside a hospital environment, with duly trained professional actors and the psychological aspect of the emotional stress involved in the performance of an evaluation, similar to that found in a real situation of emergency assistance. Therefore, the use of this methodology is a strong point of this study which deserves to be highlighted, because it presents greater validity, when compared with the application of standard questionnaires, for instance.

This methodology is widespread worldwide and employed in most Brazilian medical schools. Thus, most applicants are familiar with this type of evaluation, which is not a particularity of our institution. In spite of the greater performance in some items, the answer pattern of the applicants graduated in our institution was quite similar to that of the remaining applicants.

Simulation is an active learning methodology. In this study, it was used to evaluate the applicants, but it can also be used in medical teaching. We find it important to develop a realistic simulation standard scenario of STEMI assistance, adjusted to the particularities of the Brazilian health system, as performed in this study, because it can be reproduced in other medical schools and used in the teaching process when approaching this major cardiac disease.

An important issue that should be highlighted during the education of graduate students concerns the insertion of patients within the hierarchical network of the SUS because, in addition to establishing the adequate pharmacological treatment available in the place of care, it is important to promote an integration with the urgency and emergencies network for the prompt transfer of these patients to reference centers capable of performing the reperfusion therapy. Most students remembered to administer antiplatelet agents but, on the other hand, a significant proportion did not reinforce the need for the patient's immediate and adequate transfer, as expected in these situations.

The lack of well-organized assistance networks^14^ to refer STEMI patients from emergency care units to reference hospitals, makes the primary service doctor to have a central role in this transfer management. Only 25% of the applicants insisted on the transfer even in the non-availability of hospital bed. Therefore, it is important to raise the discussion about integrated clinical scenarios with management aspects, such as the Resolution N°. 2,077/14 of Federal Medicine Council on urgencies and emergencies, which addresses the concept of “zero vacancy”. It is extremely important that graduate students in Medicine courses understand that the treatment of this disease is time-dependent, and that it is possible to change the natural history, when treatment is set up early and, for this reason, they must insist on transfer of patients suffering from this disease to a center capable of providing reperfusion therapy, even in the absence of hospital beds.

The use of telemedicine can solve difficulties of ECG interpretation by newly graduated physicians, but this will be useless if they do not have enough knowledge on how to speed up the patient’s transfer according with the assistance flow established for each region.^15^

Simulation has already been used to assess the performance of medical students in emergency scenarios, such as STEMI assistance in other countries. However, to our knowledge, no other Brazilian study has used a similar research method.

The study showed that undergraduate students’ performance in treating simulated cases of STEMI are worse compared with other situations in which the patient is stable. For a total of 143 medical school fourth-year students, the total percentage of correct actions was 47.8% in the STEMI station, which was significantly lower in relation to the other stations (p<0.001).^16^ This fact can be observed in practice, since during the undergraduate course they end up having more contact with stable patients, while more severe patients are treated by resident physicians. Thus, simulation is considered an interesting alternative for improving emergency competencies and abilities during the undergraduate course.^17^

In an interesting study comparing simulation in small groups combined with traditional learning activities and traditional learning activities alone in 291 medical school fourth-year students for STEMI patients management, a significant improvement was observed in the performance of students in the simulation group (53.5 ± 8.9% vs. 47.4 ± 9.8%; p < 0.001), especially in relation to physical examination (48.5 ± 16.2% vs. 37.6 ± 13.1%; p < 0,02) and to diagnosis (75.7 ± 24.2% vs. 64.6 ± 25.1%; p < 0.02).^18^

Another important aspect that should be emphasized with the undergraduate student is the improvement of communication skills. A small number of applicants (34%) showed effective communication with the patient concerning the disease and the recommended treatment. This issue is often neglected in emergency care, but it is a major stage, because the patient has a right to know what is happening and the treatment strategies proposed. Besides, access to information can decrease the level of anxiety and emotional tension during an acute episode.

We highlight some limitations of this study. First, since only the applicants who were approved in the first phase of the selection process (written test) were submitted to practical evaluation, the presence of a selection bias should be considered. This may have overestimated the performance of the applicants in the selected sample and if the same evaluation had been applied to a general sample, probably the performance would have been lower than that observed. However, since in some less competitive specialties, almost all enrolled applicants were approved for the practical examination, this may have minimized this effect. Secondly, it was an evaluation applied in a single center (unicentric), but there were representatives from different regions of the country, although there was a predominance of the Southeast and Northeast regions, without an adequate sample distribution proportional to the number of graduates from different medical schools around the country. Therefore, their results cannot be extrapolated to all Brazilian territory. Thirdly, it was clarified that the fibrinolytic agent was not available in the service unit, as in the majority of these units in Brazil. Thus, immediate transfer to reperfusion therapy is indicated even in the absence of hospital beds. We agree that some differences can be observed according with the organization of the line of treatment for STEMI in each region of Brazil, like, for example, the availability of fibrinolytic agents in some of these Emergency Units. In such case, we suggest that the item *Recognizes the need of immediate transfer for reperfusion therapy* should be divided into two sub-items: administers the fibrinolytic agent and requests immediate transfer to a hospital, because either way, this patient will have to be transferred to a center capable of performing coronary intervention^19^ and, in fact, no UPA in Brazil provides this resource. Fourth, the drug heparin was not included in the checklist. This fact occurred because, during initial care in a UPA the reperfusion strategy to be adopted for the patient (fibrinolytic therapy or primary angioplasty) is unknown and, depending on the strategy assigned, the type of heparin and the dosage prescribed as first option are different and, most of the times, this medication is delayed in order to be administered in the hospital service where the reperfusion therapy will be implemented. Although they integrate the treatment, the medications, beta blockers, statins and the angiotensin converting enzyme inhibitors, have not been included, because these drugs do not need to be prescribed immediately and can be introduced throughout the first twenty-four hours. Fifth, due to restricted time for each evaluation (5 minutes), the evaluators selected some topics that they considered more relevant, such as diagnosis and therapeutic treatment, at the expense of others related with clinical history and physical examination, which is understandable, since, most of the times, few alterations are observed in the physical examination of these patients. And, finally, this tool needs external validation in other training centers.

## Conclusion

STEMI approach needs to be improved during medical undergraduate courses, highlighting practical aspects such as temporality for starting treatment and the assistance flows of these patients inside the Brazilian Unified Health System. Realistic simulation can be an adequate tool to improve these competencies.

## Supplemental Materials

* For additional information, please click here.
