# Supplementary material for: Clinical Competence in ST-segment Elevation Myocardial Infarction Management by Recently Graduated Physicians Applying for a Medical Residency Program
Source: Arq Bras Cardiol. 2020 Jan;114(1):35–44. doi: 10.36660/abc.20180309 (PMC7025295; doi:10.36660/abc.20180309)

MATERIAL SUPLEMENTAR

ANEXO A

<u>ORIENTAÇÕES AO CANDIDATO</u>

Você realizará o atendimento de um paciente adulto admitido em pronto-atendimento de Unidade Básica de Saúde.

CONTEXTO: JOÃO DA SILVA, 48 ANOS, admitido em pronto-atendimento devido dor torácica. Enfermeira do acolhimento priorizou o seu atendimento e traçou um eletrocardiograma.

HISTÓRIA CLÍNICA: Dor retroesternal em opressão de início súbito de forte intensidade 8/10 associado a náuseas. Nega irradiação e outros sintomas associados.

Antecedentes pessoais e familiares: Sem problemas de saúde. Negou usou de qualquer medicamento nas últimas 24 horas.

EXAME FÍSICO: Saturação de oxigênio: 98% (ar ambiente)

PA: 130 x 80 mmHg; FC: 88 bpm

<u>NÃO É NECESSÁRIO REALIZAR EXAME FÍSICO (Considere como normal)</u>

**TAREFAS:**
**1. DETERMINE O DIAGNÓSTICO**<br><br>**2. REALIZE O MANEJO ADEQUADO DO PACIENTE**

Atenção:

As orientações devem ser feitas verbalmente

Você dispõe de relação de medicamentos e procedimentos para a rede básica de saúde

ANEXO B

<u>ORIENTAÇÃO AOS ATORES</u>

Nome: João da Silva, 48 anos

Antecedentes: Nenhum problema de saúde. Pai e mãe sem problemas de saúde.

Características da dor:

Fácies: "de dor" " de desconforto"

Posição: "deitado no divã"

Local: ator localiza região retroesternal

Tipo: "em aperto, apertando o peito"

Forma de início: "súbito", "de uma hora para outra"

Atividade no início da dor: assistindo televisão

Tempo do início da dor: UMA HORA

Irradiação: nenhuma

Sintomas associados: "um pouco nauseado"

Manutenção da dor: "dor mantida no momento da avaliação"

Uso de medicamentos: Não usou nenhum remédio nas últimas 24 horas (Inclusive não usou Viagra)

A DOR NÃO MELHORA COM NENHUM MEDICAMENTO

Em resumo:

"Dor forte no peito há uma hora".

"Sem problemas de saúde anteriormente

"Não tomou nenhum remédio"

Intervenção do ator:

SE MÉDICO FALAR QUE O MESMO PRECISA <u>SER TRANSFERIDO ou REGULADO PARA O HOSPITAL/CENTRO DE CARDIOLOGIA</u>

<u>"Doutor, por que eu preciso ir para o hospital??"</u>

Após 10 segundos: <u>"Doutor e se não tiver vaga ??"</u>

ANEXO C

LISTA DE CHECAGEM (CHECKLIST)

**1**	Solicita monitorização<br>(monitor ECG contínuo)	Ë SIM<br>(0,5 pontos)	-	Ë NÃO<br>(0,0 pontos)
**2**	Solicita acesso venoso<br>(acesso periférico)	Ë SIM<br>(0,5 pontos)	-	Ë NÃO<br>(0,0 pontos)
**3**	Avalia a duração da dor<br>(tempo de início da dor)	Ë SIM<br>(1,0 pontos)	-	Ë NÃO<br>(0,0 pontos)
**4**	Reconhece IAM com supradesnivelamento de ST<br>(IAM com supra/ Infarto com supra)	Ë SIM<br>(2,0 pontos)	Ë PARCIAL<br>(1,0 pontos)	Ë NÃO<br>(0,0 pontos)
**5**	Realiza AAS<br>(aspirina, ácido acetilsalicílico)	Ë SIM<br>(0,5 pontos)	-	Ë NÃO<br>(0,0 pontos)
**6**	Realiza Clopidogrel<br>(Plavix®, Brilinta®, Effient®, Ticagrelor, Prasugrel)	Ë SIM<br>(0,5 pontos)	-	Ë NÃO<br>(0,0 pontos)
**7**	Realiza nitrato sublingual<br>(Isordil, Sustrate, Isossorbida)	Ë SIM<br>(0,5 pontos)	-	Ë NÃO<br>(0,0 pontos)
**8**	Realiza morfina endovenosa<br>(Dimorf®)	Ë SIM<br>(0,5 pontos)	-	Ë NÃO<br>(0,0 pontos)
**9**	Reconhece necessidade de transferência imediata para terapia de reperfusão<br>(angioplastia, ACTP, cateterismo, trombolítico, estreptoquinase, alteplase, Actylise® tenecteplase, Metalyse®)	Ë SIM<br>(2,0 pontos)	Ë PARCIAL<br>(1,0 pontos)	Ë NÃO<br>(0,0 pontos)
**10**	Habilidade de comunicação com o paciente<br>Explica para o paciente sobre doença e tratamento de forma adequada<br>(vaso do coração entupiu e precisa de remédio ou cateterismo para desobstruir)	Ë SIM<br>(1,0 pontos)	Ë PARCIAL<br>(0,5 pontos)	Ë NÃO<br>(0,0 pontos)
**11**	Insiste na transferência para hospital mesmo sem vaga (VAGA ZERO)	Ë SIM<br>(1,0 pontos)	-	Ë NÃO<br>(0,0 pontos)
**12**	Espera marcadores de necrose (CK-MB, troponina) para definição da conduta	Ë SIM<br>(-2,0 pontos)	-	Ë NÃO<br>(0,0 pontos)

ANEXO D

ORIENTAÇÕES AOS AVALIADORES
1) Não comunicar com o candidato.<br>2) Entregar o ECG para o candidato.<br>3) Não há derivações adicionais: V3R, V4R, V7 e V8.<br>4) O candidato não precisa monitorizar ou pegar acesso venoso periférico, mas somente solicitar tais procedimentos.<br>5) No Item 4.0, considera-se a resposta correta: IAM com supradesnível do segmento ST ou IAM com supra ou Síndrome Coronariana com supra ST, considera-se como resposta parcial o diagnóstico de infarto, porém não especifica com SUPRA. Se o candidato falar Infarto de parede anterior também pontua como parcial.<br>6) O candidato precisa falar o nome dos medicamentos, não será avaliado a dose correta dos mesmos.<br>7) No item 9.0, considera-se a resposta correta: transferência imediata para o hospital para realização de terapia de reperfusão (trombolítico ou cateterismo ou angioplastia), considera-se resposta parcial se reconhece a necessidade de transferência, porém não relata a intervenção necessária.<br>8) A administração de oxigênio é indiferente para esta estação.<br>9) Se candidato questionar sobre disponibilidade de trombolítico ou angioplastia, ou outros medicamentos e intervenções na Unidade, falar para o mesmo avaliar a RELAÇÃO DE MEDICAMENTOS E PROCEDIMENTOS PARA A REDE BÁSICA disponibilizada na sala.<br>10) Outras medicações como heparina, enoxaparina, betabloqueador, estatina, inibidor de ECA não estão sendo avaliados nesta estação, pois os dois primeiros dependem do tipo de tratamento instituído (trombolítico vs. angioplastia) e os demais deverão ser iniciados preferencialmente nas primeiras 24 horas, mas não necessariamente de imediato.<br>11) No item 10. 0 deverá ser avaliada a comunicação do candidato com o paciente, o candidato deverá explicar em linguagem clara e simples a doença e o tratamento proposto, exemplo: “tem uma artéria do coração entupida, isto causou o infarto, você precisará realizar um procedimento para desentupir esta artéria. Se explicar somente a doença ou tratamento considera-se resposta parcial.<br>12) No item 11.0 somente será pontuado se o candidato insistir na transferência mesmo se não houver VAGAS, após a pergunta do ator sobre a disponibilidade de vagas. Também será pontuado se referir a expressão “VAGA ZERO”.<br>13) No item 12.0, se candidato pedir exames porém isto não interferir na sua conduta de instituir tratamento e transferir imediatamente considera-se como NÃO. Somente pontua-se negativamente (-2) se ficar esperando o resultados de exames para definição desta conduta.

ANEXO E

RELAÇÃO DE MEDICAMENTOS PARA A REDE BÁSICA DE SAÚDE (SUS)	
**A**	Ácido acetilsalicílico 100 mg, comprimido
	Ácido fólico 5 mg, comprimido
	Amiodarona 150 mg, ampola, injetável
	Amoxicilina 500 mg, comprimido
	Anlodipino 10 mg, comprimido
	Atenolol 50 mg, comprimido
**B**	Benzilpenicilina benzatina , 1200000 UI, injetável
	Bromoprida 10 mg, ampola, injetável
**C**	Captopril 25 mg, comprimido
	Carvedilol 12,5 mg, comprimido
	Cefalexina 500 mg, comprimido
	Ceftriaxona 1 g, injetável
	Clopidogrel 75 mg, comprimido
**D**	Dipirona 500 mg/ml, solução oral
**E**	Enalapril 20 mg, comprimido
	Escopolamina 6.67 mg/ml, solução injetável
	Espironolactona 25 mg, comprimido
**F**	Fenoterol 5mg/ml solução inalante gotas
	Fluoxetina 20 mg, cápsula
	Furosemida 40 mg, comprimido
**G**	Glibenclamida 5 mg, comprimido
**H**	Heparina não fracionada injetável SC (5000 UI/ml)
	Hidroclorotiazida 25 mg, comprimido
	Hidrocortisona 100 mg, ampola injetável
**I**	Ibuprofeno 600 mg, comprimido
	Insulina humana regular 100 UI/ml injetável
	Insulina humana NPH 100 UI/ml, injetável
	Isossorbida, dinitrato, 5 mg comprimido
	Isossorbida, mononitrato, 10 mg comprimido
**L**	Levotiroxina 50 mcg, comprimido
	Losartana 25 mg, comprimido
**M**	Metformina 500mg, comprimido
	Metoclopramida 10 mg, injetável
	Midazolam 15 mg, ampola, injetável
	Morfina 10 mg, injetável
**O**	Omeprazol 20 mg, cápsula
**P**	Paracetamol 500 mg, comprimido
	Prednisona 20 mg, comprimido
	Propranolol 40 mg, comprimido
**S**	Sais para reidratação oral, envelope
	Salbutamol, frasco aerossol
	Sinvastatina 20 mg, comprimido
	Solução fisiológica 0,9%, 10 ml, 100 ml, 500 ml, 1000 ml

RELAÇÃO DE PROCEDIMENTOS PARA A REDE BÁSICA DE SAÚDE (SUS)	
**A**	Abocath e scalp
**C**	Cardioversor elétrico
	Cateter de oxigênio
**E**	Eletrocardiograma convencional de 12 derivações
	Exames bioquímicos gerais (glicemia, hemograma, creatinina, sódio, potássio, CK-MB, urina rotina)
**G**	Glicosimetria capilar
**M**	Monitor cardíaco

**O**	Oxímetro de pulso
**R**	Radiografia de tórax, abdome e membros
**S**	Sondagem vesical
	Sondagem nasogástrica
**T**	Tubo oro traqueal 6.0, 7.0, 7,5 e 8,0

ANEXO F

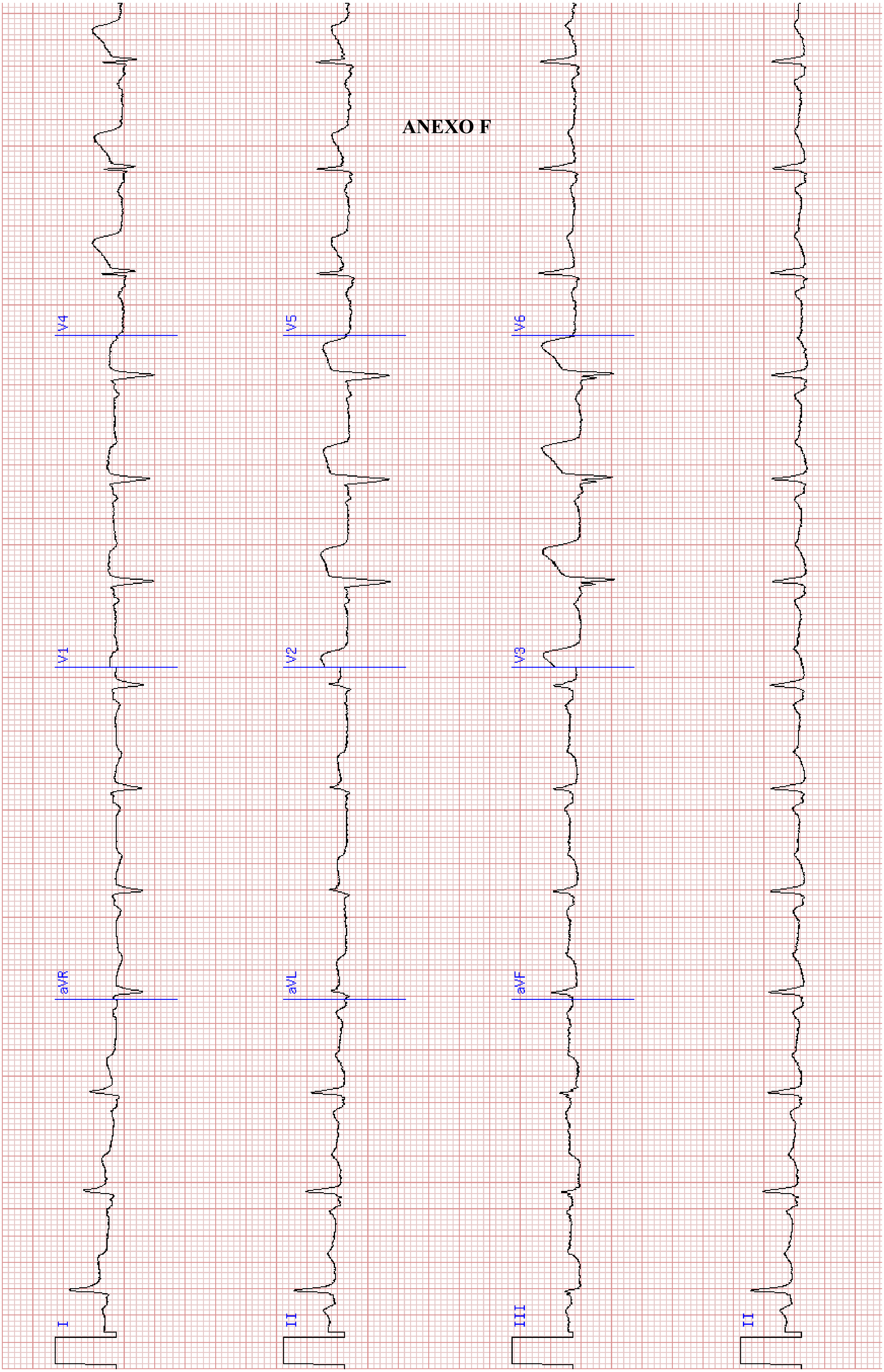

Supplement: * — For additional information, please click here. [file abc-114-01-0035-suppl01.pdf]
